# Case Report: Treatment of Delusions of Theft Based on the Assessment of Photos of Patients' Homes

**DOI:** 10.3389/fpsyt.2022.825710

**Published:** 2022-03-17

**Authors:** Daiki Ishimaru, Hideki Kanemoto, Maki Hotta, Yuma Nagata, Yuto Satake, Daiki Taomoto, Manabu Ikeda

**Affiliations:** ^1^Department of Medical Technology, Osaka University Hospital, Suita, Japan; ^2^Department of Psychiatry, Osaka University Graduate School of Medicine, Suita, Japan

**Keywords:** case report, delusion of theft, Alzheimer's disease, assessment, environmental factor

## Abstract

**Background:**

The occurrence of behavioral and psychological symptoms of dementia is affected by individualized context. However, details regarding delusion of theft have been poorly documented. This report describes a useful assessment to understand the environmental context of delusion through two cases of Alzheimer's disease (AD).

Familial interview was conducted to assess the phenomenological features. Photos of patients' homes were used to increase the assessment accuracy and check the individualized environmental contexts; this is known as Photo Assessment of Living Environment (PA-LE).

**Case Description:**

Case 1 was of an 88-year-old woman whose Mini-Mental State Examination (MMSE) score was 23/30. She believed that one neighbor stole her wallet and stored it on a shelf in the living room. She sometimes placed it in other places, such as under the bed as safekeeping. The delusion often occurred when getting ready to go shopping. PA-LE confirmed that the room and shelf were not cluttered, although the incorrect storage place seemed to be hard-to-find.

Case 2 was of a 78-year-old woman. The MMSE score was 20/30. She believed that some neighbors stole her garden items. The delusion was limited to her garden, yet the items were varied. Auditory hallucinations exacerbated her belief that the neighbors intruded the garden. PA-LE confirmed that the garden was cluttered with several duplicated items. Moreover, the patient inaccurately remembered the condition of the garden.

Non-pharmacological approaches were tailored to the patients' environmental and psychological states, referring to the interview and PA-LE. This included environmental adjustment or increasing self-esteem. Antipsychotics were also prescribed. Environmental and psychological triggers of delusion were improved by the interventions, and the patients had uneventful courses without active delusions.

**Conclusion:**

Evaluating patients' homes using photos could detect the environmental context of delusion of theft among patients with AD and assist in the management.

## Introduction

Behavioral and Psychological Symptoms of Dementia (BPSD) are clinically important symptoms in people with dementia and frequently occur in their clinical course (1). BPSD often cause negative outcomes, including early hospital admission, significant care burden, and decrease in the quality of life (2–4). In the non-pharmacological intervention as first-line treatment for BPSD, understanding the mechanism that caused each of the BPSD is of critical importance (5). Notably, the occurrence of BPSD is well-known to be involved in highly individualized contexts (6), such as the living environment or relationship with the caregiver. Generally, it would be difficult to accurately assess these individualized contexts using only the existing standard assessment scale (7).

Delusion of theft is one of the most common BPSD in patients with Alzheimer's disease (AD). The mechanism of delusion of theft has been well-identified (8); however, details of individualized contexts and specific methods have not yet been thoroughly documented. As one example, it is conceivable that cluttered rooms could induce the failure experience of misplacing things, potentially increasing the risk for delusions of theft. However, such environmental contexts have been too highly individual to implement a general assessment or intervention.

This case report preliminarily shows an overview of the new assessment method through interviews using photographs of the homes of two AD inpatients. We believe that this method will be useful for visually assessing individualized environmental contexts and describing the phenomenological features of delusions of theft to develop individualized intervention plans.

## Case Description

Parts of personal information were anonymized to protect patients' privacy. Written informed consent from both patients was obtained for the publication of this case report.

The Japanese version of the 12-item Neuropsychiatric Inventory (NPI-12) was used to assess BPSD (9). The NPI-12 includes 12 domains: delusions, hallucinations, agitation/aggression, depression/dysphoria, anxiety, elation/euphoria, apathy/indifference, disinhibition, irritability/lability, motor disturbance, nighttime behaviors, and appetite/eating. A composite score of all domains was calculated by multiplying the frequency and severity within each domain as follows: 1–4 (4 = most frequent) and 1–3 (3 = most severe), respectively. The total score ranges from 0 to 144, with higher scores indicating more severe neuropsychiatric symptomology.

We conducted the new method of “Photo Assessment of Living Environment (PA-LE)” to identify environmental context involved in delusion of theft. The photos of the outdoors, indoors, and home appliances were prepared by the patients' families according to the photographic place guidance. The guidance consisted of seven sheets describing how to photograph the exterior and indoor environment, including the bedroom, living room, kitchen, and closet, and home appliances, such as the refrigerator and washing machine. The guidance also included the house layout. Additionally, the places and objects that seemed to be associated with the delusion of theft were photographed. Familial interviews were conducted to identify the phenomenological features of the delusion of theft. In this interview, questions associated with the delusion of theft, including the frequency, person, item, time, and location were asked (Table 1), while observing the photo of the patient's home. Photos depicting the state of their homes were used to increase interview accuracy and not overlook the important findings. We also assessed the psychosocial aspects such as self-esteem, daily life at home, or life history from the patient or the family.

**Table 1:** Delusion features assessed using interviews and photographs.

	**Case 1**	**Case 2**
Age (years)	88	79
Sex	Female	Female
Diagnosis	AD^†^	AD^†^
Duration of delusion (years)	4	1
MMSE^‡^	23/30	20/30
Living arrangement	Alone	With daughter and granddaughter
**Delusion of theft features**
Frequency	Unknown	Almost daily
Who stole	One specific neighbor	Some neighbors
What was stolen	Wallet and passbook	Various garden items
Where the delusion occurred	Living room	Garden
When the delusion occurred	Unspecified	Midnight
Why the patient thought that their objects are stolen	Inability to find the wallet before going shopping	Hearing digging sounds and footsteps in the middle of the night
How the patient dealt with the theft	Complaints to the neighbor, police notification, surveillance cameras installed, and specific windows were blockaded	Neighbor notification, police notification, surveillance cameras installed, and garden fences were added

† *Alzheimer's disease*.

‡ *Mini-Mental State Examination*.

Non-pharmacological and pharmacological treatments, i.e., antipsychotics, were provided. Non-pharmacological treatment involved identifying several targets tailored to the patient's psychological and environmental state, while also contextualizing the delusion of theft based on a comprehensive perspective such as the interview assessments, PA-LE, and the neuropsychological tests. Non-pharmacological treatments to address these targets were planned and implemented to (a) increase self-esteem, (b) decrease the triggers of delusion, (c) replace the time spent on delusional thinking with other activities, and (d) provide pleasure in their daily lives. Table 2 shows the details of contents and background of planning in non-pharmacological intervention.

**Table 2:** The content and background of non-pharmacological intervention.

**Case 1**	**Case 2**
***(a) Increase self-esteem*** Avoiding identification of her failures such as losing valuables was encouraged, since she tended to have high levels of self-reliance from the familiar interview. We also offered her family the specific place that they should put her valuables back based information of the PA-LE (Figures 1C,D)	***(a) Increase self-esteem*** Based on the interview with her and family, the change in household roles was likely to affect her self-esteem. We proposed her family that they gave her several roles in the family. We also advised the family to avoid denying her false beliefs, in order not to decrease her self-esteem
***(b) Decrease the triggers of delusion*** Inability to find the wallet before going shopping was inferred as one of the triggers of delusion by the interview of phenomenon regarding delusion. As such, we encouraged the family to get ready and go shopping with her	***(b) Decrease the triggers of delusion*** The cluttered garden was presumed to exacerbate her failure experience that she could not find own properties based on information of the PA-LE. Based on the information of PA-LE, we proposed to her family that they reduced the objects, especially duplicate items and items that she has not been using for a while in the garden (Figure 2C) and limited the specific storage place of garden items (Figure 2D) in consultation with her
***(c) Replace the time spent on delusional thinking with other activities*** Based on the familial interview of daily life, she tended to have less opportunities of engaging in meaningful activities since much of housework was done by others. We encouraged the family to involve her in several housework such as washing or sweeping, tasks that she could perform.	***(c) Replace the time spent on delusional thinking with other activities*** Based on the interview regarding the phenomenon of delusion, her delusion tended to occur with auditory hallucination in the middle of the night. We encouraged the family to increase her daytime activity such as attending an adult day care, in order to decrease night awakening
***(d) Provide pleasure in their daily lives*** She actively engaged and enjoyed interaction with other inpatients of same age during her hospital stay. We encouraged her and her family to talk to individuals of the same age as part of group therapy in an adult day care after discharge	***(d) Provide pleasure in their daily lives*** She took care of her garden at home and enjoyed a horticulture program during hospital stay. We encouraged her to engage in gardening more proactively and safely as a leisure activity, while decreasing the duplicated and not-necessary items and limiting the storage place of garden items

### Case 1

An 88-year-old woman was admitted to our psychiatric ward for delusions. She lived alone, and much of the housework was done by her family. She had engaged in volunteer activities, such as helping the local community, for over 20 years. However, she was unable to continue to volunteer due to community changes and the chairman's replacement that resulted in hostility against the neighbors. Her self-esteem likely decreased as a result. Four years ago, she began to suspect that her properties were stolen by a neighbor. This led to increasing problems with the neighbors and involvement of the police.

**Figure 1:**
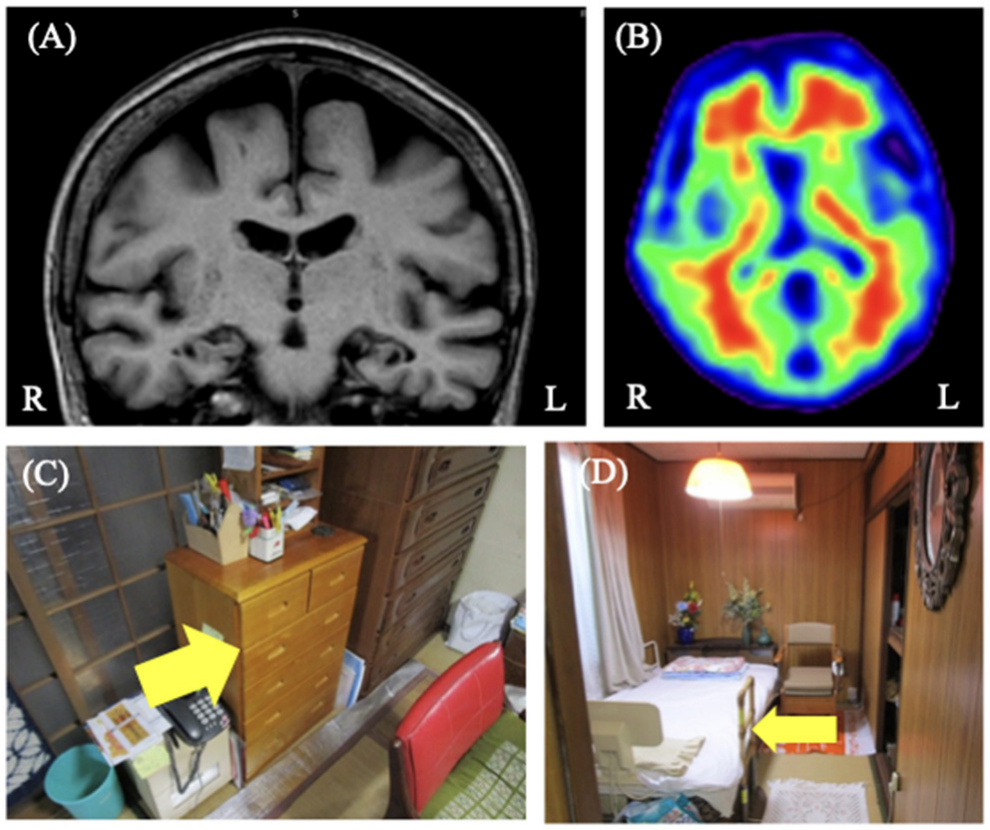
Case 1 images. **(A)** Brain magnetic resonance T1-weighted image showed diffuse cortical atrophy but relative sparing of the medial temporal lobes with bilateral hippocampal sulcus remnant. **(B)** Florbetapir 18 positron emission tomography showing positive results. **(C,D)** These are the images of the patient's home. She usually stored her valuables such as wallet in yellow arrowed shelf **(C)**. She sometimes hid her valuables under the yellow arrowed bed **(D)**. We proposed to her family that they put her valuables back to usually stored place if they found them in unusual places.

On admission, the patient's Mini-Mental State Examination (MMSE) score was 23/30. The logical memory scores in the Wechsler Memory Scale-Revised (WMS-R) were 2 for immediate and 0 for delayed recall. The Frontal Assessment Battery (FAB) score was 17/18. Addenbrooke's Cognitive Examination III (ACE-III) score was 74/100 and sub-domain scores were as follows: attention, 16/18; fluency, 11/14; visuospatial, 16/16; memory, 10/26; and language, 21/26. Neuropsychological tests mainly revealed partial disorientation to time and place with mild to moderate amnesia. The total NPI-12 score was 24/144. The rated symptoms and their respected scores were as follows: delusions, 12; hallucinations, 2; agitation/aggression, 4; elation/euphoria, 3; and night-time behaviors, 3. Brain magnetic resonance imaging (MRI) showed diffuse cortical atrophy prominently in the bilateral parietal but relatively sparing the medial temporal lobes with bilateral hippocampal sulcus remnant (Figure 1A). Florbetapir F 18 positron emission tomography (PET) showed amyloid depositions mainly in the frontal cortex (Figure 1B). In the cerebrospinal fluid testing, phosphorylated tau at epitope 181 (p-tau) and total tau (t-tau) were 103 and 830 pg/mL, respectively. These results were suggestive of AD pathology (10).

The features of delusions were assessed through interviews and photographs of the patient's home (Table 1). Familial interviews revealed that she strongly believed that one specific neighbor stole her wallet and passbook. The delusion often occurred when she could not find her wallet in the shelf while getting ready to go shopping. The time of the day describing the theft was unspecified and the frequency of delusions was unknown because she lived alone. The delusional misidentification syndrome was not exhibited. The items she accused of being stolen were usually stored on a shelf in the living room, while she sometimes kept these items in incorrect places (where she thought were safer), such as under the bed. Figure 1 shows the photos of the rooms and places associated with these delusions through PA-LE (Figures 1C,D). The photos revealed that the shelf and living room she frequently used were not cluttered, and it seemed to be easy to find her wallet. In contrast, the incorrect storage places, such as under the bed, might have been hard to find the wallet in, although the place was not cluttered.

Risperidone (0.5 mg/day) was prescribed. A few days later, delusional thinking and agitation improved relatively; however, the original delusion of theft remained for about 7 weeks from the start of an atypical antipsychotic till discharge. For further management of delusion, upon discharge, we provided her caregivers with instructions regarding non-pharmacological interventions which included the following (Table 2): (a) To increase the patient's self-esteem, avoid pointing out failures, such as losing valuables, and consider putting them back in their usual place by the family based on the information of the PA-LE (Figures 1C,D). (b) To decrease triggers of delusion, encourage the family to get ready and go shopping with her. (c) To decrease the time spent for delusional thinking, leave her several housework such as washing or sweeping she could perform. Finally, (d) to increase pleasure in her daily life, encourage talking with individuals of the same age as part of group therapy in an adult day care.

The patient had an uneventful course without active delusions during the post-discharge period. The guidance we provided altered the family's attitude toward her and increased her daily activities, such as engagement in leisure activities and attending an adult day care. Her family also tried to help her with shopping to avoid her failure experience. She spent much of her daily time in arranging the garden as a pleasurable role. One year after discharge, she continued living alone without major problems.

### Case 2

A 78-year-old woman was admitted to our psychiatric ward for treatment of delusions. She lived with her oldest daughter and granddaughter. She had worked in a public hall, where she interacted with local people. In the past 10 years, she lost numerous relatives, including her husband and sister. Her daughter had also recently changed her long-term job. These events led her to feel anxious. In the past year, her daughter and granddaughter performed most of the housework because the patient suffered an upper arm fracture. Since then, she began to suspect that her garden items, which she used for leisure activities, were stolen by the neighbors. This led to increasing problems with the neighbors and involvement of the police.

**Figure 2:**
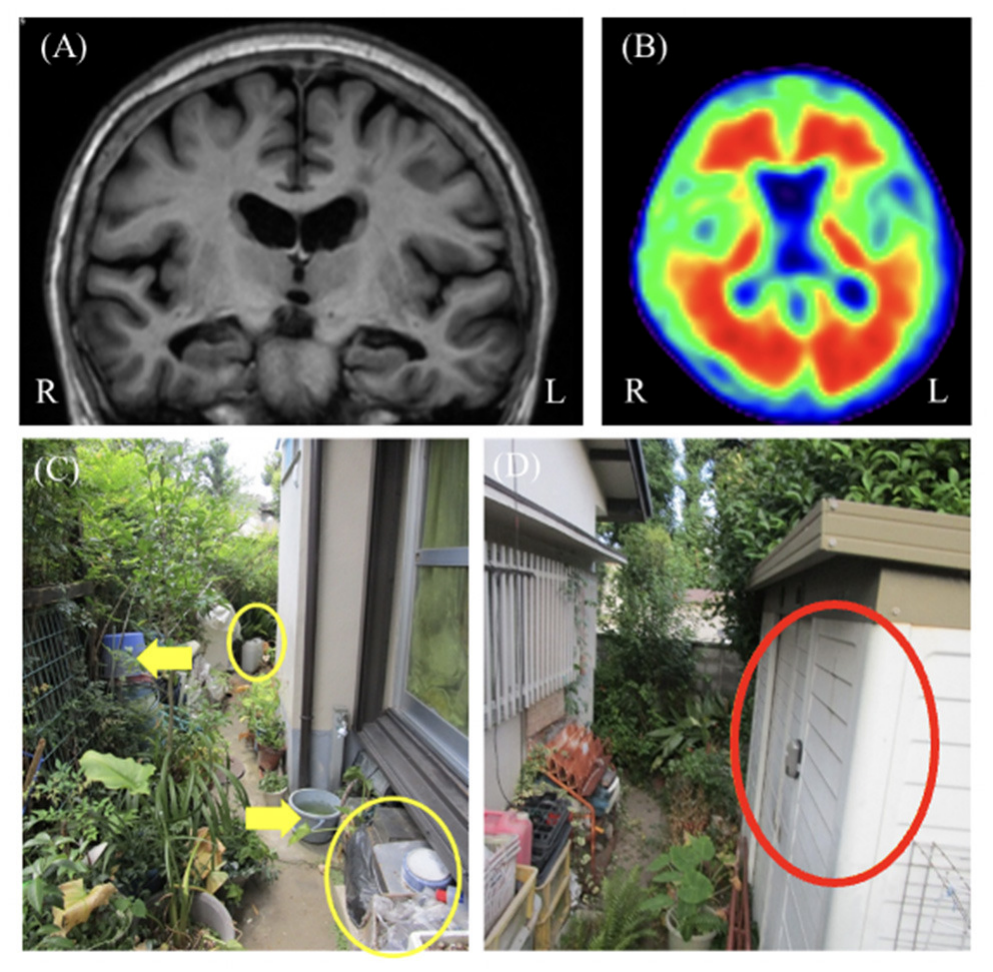
Case 2 images. **(A)** Brain magnetic resonance T1-weighted image showed bilateral hippocampal atrophy. **(B)** Florbetapir positron emission tomogrpahy showing positive results. **(C,D)** These images show the patient's cluttered garden. The yellow circled items were the examples of things she has not been used in a while. The yellow arrowed ones were the examples of duplicated items. We proposed to her family that they reduced these items after consulting with her **(C)**. The red circled storage was one of the several storage places of her garden items. We proposed to her and the family that they limitedly stored the garden items in just this place **(D)**.

On admission, her MMSE score was 20/30. The logical memory scores in the WMS-R were 3 for immediate recall and 0 for delayed recall. The FAB score was 9/18. ACE-III score was 65/100 and sub-domain scores were as follows: attention, 14/18; fluency, 8/14; visuospatial, 14/16; memory, 6/26; and language, 23/26. Neuropsychological tests mainly revealed partial disorientation to time and place with mild to moderate amnesia, decreased attention function, and constructional disability. The total NPI-12 score was 58/144. The rated symptoms and their respective scores were as follows: delusions, 12; hallucinations, 12; depression, 8; anxiety, 8; apathy, 8; disinhibition, 6; and night-time behaviors, 4. Brain MRI showed diffuse cortical atrophy, which was relatively prominent in the bilateral hippocampal and parietal lobes (Figure 2A). Florbetapir PET showed amyloid depositions more in the posterior temporal and occipital lobes (Figure 2B). In the cerebrospinal fluid testing, phosphorylated tau at epitope 181 (p-tau) and total tau (t-tau) were 64 and 316 pg/mL, respectively. These results were suggestive of AD pathology (10). Neurological examination was unremarkable. The patient had a history of hypertension, dyslipidemia, and left cerebral aneurysm. She was diagnosed with probable AD according to the National Institute on Aging-Alzheimer's Association 2011 criteria (11).

The features of delusions were assessed through interviews and photos (Table 1). The familial interview revealed that the patient believed that some of her neighbors stole her garden items, such as flowerpots and trowels. This delusion was only associated with her garden, whereas the type of items suspected to be stolen varied. Her family also reported that she experienced hearing noises from the garden, such as digging sounds and footsteps in the middle of the night. These were not proven to be accurate, and the patient did not have any hearing impairment. The auditory hallucinations were likely to exacerbate her belief that some neighbors intruded into her garden. Therefore, the delusion often occurred at midnight. The delusional misidentification syndrome was not exhibited. Figure 2 shows the photos of the garden and items associated with these delusions through PA-LE (Figures 2C,D). The photos revealed that the garden was cluttered and there were many items with duplicates. There were also several storage places of her garden items. The patient was unable to accurately remember their location and the quantity of the garden items.

Risperidone (0.5 mg/day) was prescribed. A few days later, the patient developed mild upper limb rigidity, whereas her mood and delusional thinking remained mostly unchanged. It was continued as an outpatient treatment to monitor the progress of the treatment effect. The original delusion of theft remained for 5 days from the start of an atypical antipsychotic till discharge. For the further management of delusion, upon discharge, we provided her caregivers the with the following instructions regarding non-pharmacological interventions (Table 2): (a) To increase her self-esteem, give her several roles in the family, and avoid denying her false beliefs. (b) To decrease the triggers of delusion, reduce the objects such as duplicate items and items that she has not been using for a while in the garden (Figure 2C) and limit the specific storage place of garden items (Figure 2D) with her consultation. (c) To decrease the time spent on delusional thinking, increase her daytime activity such as attending the adult day care, in order to decrease night awakening. Finally, (d) to improve pleasure in daily life, encourage her and the family to engage in gardening more proactively and safely as a leisure activity, while decreasing the duplicated and not-necessary items and limiting the storage place of garden items. After discharge, the patient lived in both her oldest and second daughter's homes in turns.

The patient had an uneventful course without active delusions in her second daughter's home. However, a new delusion that her granddaughter stole her item occurred in the oldest daughter's home, although her original delusion was not exacerbated. According to the guidance we provided, the cluttered garden was partially improved by herself and her family member, and she attended the adult day care. There was also the patient's comment “Now that we've cleaned up some of the stuff in the garden, it's not stolen at all.” One year after discharge, former behavioral problems, such as those with the neighbor or police notifications, did not occur. Delusions of theft seemed to have reduced to tolerable or acceptable levels.

## Discussion

This case report described two patients with AD and delusions of theft who were evaluated through familial interviews and photographs of their homes, revealing the phenomenological features of delusion of theft with the living environments. Moreover, we noted that several individualized features of the environments, such as a non-unified and hard-to-find storage space, or cluttered places, may have partially exacerbated the false belief that the patients' own properties were stolen by someone. In addition, the non-pharmacological interventions that were tailored to the patients' environmental and psychological states were likely to show partial improvement of delusion, assisting them to continue living in the community.

Delusion of theft is caused by interactions among various factors, including gender, cognitive deficit, loneliness, negative feelings, defense mechanisms, and brain dysfunction (8, 12, 13). Understanding the phenomenological features with several individualized environmental contexts found through our report may assist in interpreting the complex personal experiences of these patients and implementing individualized non-pharmacological interventions. Tible et al. (14) reported that qualitative phenomenological approach could help interpret the behavioral symptoms of patients with dementia. Considering that what was thought to be stolen and the situation around it were relatively limited, the failure experience associated with memory impairment may have been an important trigger in the patient in Case 1 of our report. Symptoms in the patient in Case 2 may have been partially caused by environmental factors, such as disorganized or duplicated items, although auditory hallucinations could have also contributed to these delusions. Additionally, the two AD patients seemed to have responded positively to the non-pharmacological interventions that were tailor-made based on the assessment of their individual contexts, although the effect of the non-pharmacological intervention could not be clearly distinguished from that of the pharmacological intervention. A possible explanation for this result is that the intervention altered several environmental and psychological triggers associated with delusion of theft according to each individual's context of events. In recent years, environmental factors, such as home environment or environmental stimulation, have received considerable attention as modifiable factors related to BPSD for the development of effective interventions (15, 16).

A qualitative study showed that interviews with photos can help understand the daily life of people with dementia (17). This supports the concept used in our case report. The photos of patients' homes provide more information about the actual state of their daily life than the interview assessments. Interestingly, Ramsdell et al. (18) have reported that interview assessments resulted in an underestimation of the degree of identified problems of people with dementia compared with home-visiting assessments. They also indicated that the potential risk for problems in daily life can be checked through observation of the patient's living environment. This indication agrees with our experience with PA-LE. For instance, if there had been no visual data of the patients' homes based on PA-LE, the hard-to-find storage place or significant cluttering in the garden, such as those that would potentially exacerbate the failure experience associated with memory impairment, would not have been confirmed in detail. Incorporating visual information of the patients' living environments into the general assessment of delusions of theft likely contributed to a more accurate understanding of the individualized environmental context for the patients' delusions.

To our knowledge, this case report is the first to reveal the phenomenological features of delusions of theft using individualized environmental contexts based on familial interviews and photographs of patients' homes. The findings of this case report will help understand the individualized environmental contexts for not only delusions but also other BPSD and provide effective management tailored to the environmental and psychological state of each patient.

In conclusion, interview assessments with photographs of the patients' homes helped identify the phenomenological features of the environmental contexts for the delusions of theft in patients with AD. PA-LE may be useful for understanding the individualized environmental context of delusions. Future prospective studies involving larger populations are required to reveal the effects of environmental factors on delusions of theft. A careful design that could clearly identify how much improvement is due to non-pharmacological intervention instead of pharmacological therapy will be useful to determine an effective intervention tailored according to these factors.

## Data Availability Statement

The raw data supporting the conclusions of this article will be made available by the authors, without undue reservation.

## Ethics Statement

Written informed consent was obtained from the individual(s) for the publication of any images or data included in this article. Parts of personal information were anonymized to protect patients' privacy.

## Author Contributions

DI and YS: treated the patient during admission, while MI, YS, and DT: conducted the outpatient treatment. DI: wrote the first draft. HK, MH, YN, YS, DT, and MI: offered advice for interpretation of the result and participated in the discussion. All authors contributed to the article and approved the submitted version.

## Funding

This work was supported by the JSPS KAKENHI Grant-in-Aid for Young Scientists (no. 21K17528), AMED under Grant Number JP21dk0207056, and AMED under Grant Number 19de0107001h0001.

## Conflict of Interest

The authors declare that the research was conducted in the absence of any commercial or financial relationships that could be construed as a potential conflict of interest.

## Publisher's Note

All claims expressed in this article are solely those of the authors and do not necessarily represent those of their affiliated organizations, or those of the publisher, the editors and the reviewers. Any product that may be evaluated in this article, or claim that may be made by its manufacturer, is not guaranteed or endorsed by the publisher.
